# Exogenous dsRNA Induces RNA Interference of a Chalcone Synthase Gene in *Arabidopsis thaliana*

**DOI:** 10.3390/ijms23105325

**Published:** 2022-05-10

**Authors:** Nikolay N. Nityagovsky, Konstantin V. Kiselev, Andrey R. Suprun, Alexandra S. Dubrovina

**Affiliations:** Laboratory of Biotechnology, Federal Scientific Center of the East Asia Terrestrial Biodiversity, Far Eastern Branch of the Russian Academy of Sciences, 690022 Vladivostok, Russia; nityagovskii@biosoil.ru (N.N.N.); kiselev@biosoil.ru (K.V.K.); suprun@biosoil.ru (A.R.S.)

**Keywords:** exogenous dsRNA, gene silencing, RNA interference, small RNAs, plant foliar treatment, plant gene regulation

## Abstract

Recent investigations have shown the possibility of artificial induction of RNA interference (RNAi) via plant foliar treatments with naked double-stranded RNA (dsRNA) to silence essential genes in plant fungal pathogens or to target viral RNAs. Furthermore, several studies have documented the downregulation of plant endogenous genes via external application of naked gene-specific dsRNAs and siRNAs to the plant surfaces. However, there are limited studies on the dsRNA processing and gene silencing mechanisms after external dsRNA application. Such studies would assist in the development of innovative tools for crop improvement and plant functional studies. In this study, we used exogenous gene-specific dsRNA to downregulate the gene of chalcone synthase (CHS), the key enzyme in the flavonoid/anthocyanin biosynthesis pathway, in Arabidopsis. The nonspecific *NPTII*-dsRNA encoding the nonrelated neomycin phosphotransferase II bacterial gene was used to treat plants in order to verify that any observed effects and processing of *AtCHS* mRNA were sequence specific. Using high-throughput small RNA (sRNA) sequencing, we obtained six sRNA-seq libraries for plants treated with water, *AtCHS*-dsRNA, or *NPTII-*dsRNA. After plant foliar treatments, we detected the emergence of a large number of *AtCHS*- and *NPTII*-encoding sRNAs, while there were no such sRNAs after control water treatment. Thus, the exogenous *AtCHS*-dsRNAs were processed into siRNAs and induced RNAi-mediated *AtCHS* gene silencing. The analysis showed that gene-specific sRNAs mapped to the *AtCHS* and *NPTII* genes unevenly with peak read counts at particular positions, involving primarily the sense strand, and documented a gradual decrease in read counts from 17-nt to 30-nt sRNAs. Results of the present study highlight a significant potential of exogenous dsRNAs as a promising strategy to induce RNAi-based downregulation of plant gene targets for plant management and gene functional studies.

## 1. Introduction

RNA interference (RNAi) is a natural regulatory mechanism that functions in plants and other eukaryotes to defend them against pathogens and transposable elements and to downregulate the expression of endogenous protein-coding genes [1,2,3]. RNAi involves sequence-specific degradation of target mRNAs or translation inhibition induced by small RNAs (sRNAs), which are generally divided into two main categories, microRNA (miRNAs) and small interfering RNA (siRNAs) pathways [4,5]. The siRNA-mediated RNAi pathway is generally based on the recognition and processing of dsRNA precursors that are converted into siRNAs, i.e., 20–24-nucleotide (nt)-long RNA duplexes, by a ribonuclease DICER. These siRNAs are then incorporated into the RNA-induced silencing complex (RISC) to sequester the single-stranded siRNA guide strand and induce gene silencing in a sequence-specific manner via cleavage, destabilization, or translation repression of homologous mRNAs.

It is known that different sRNAs that are generated in plant cells can spread throughout the plant via the plant vascular system and can be transferred to plant pathogens via extracellular vesicles [5,6,7]. Recent studies have indicated that plants can uptake and process externally applied synthetic dsRNAs [8,9,10,11,12,13] or siRNAs [12,14,15,16,17] that have been artificially designed and supplied to activate RNAi in plants and plant pathogens and silence target genes in a sequence-specific manner. Both plants and infecting pathogens were capable of dsRNA uptake, and this eventually triggered RNAi-mediated silencing of the pathogen virulence-related genes or targeted virus RNA for degradation [6,8,11,18,19,20]. These dsRNAs were capable of systemic spreading within the plant and were transferred to the pathogens/pest. Currently, external dsRNA or siRNA application is being developed as a promising tool for plant pathogen protection and is currently termed spray-induced gene silencing (SIGS) [21,22,23]. It appears that the application of dsRNA or siRNAs to external plant surfaces is a promising instrument of plant RNA-based “vaccination” that can become an alternative to transgenic plants and virus-induced gene silencing (VIGS) [22,24,25,26].

Currently, there is a high number of studies reporting on successful external dsRNA application to the plant surfaces for RNAi induction and plant pathogen protection (reviewed in [25,26,27]). However, there is a limited number of investigations reporting on the silencing of plant endogenous genes after exogenous dsRNA or siRNA application. According to our literature analysis, there are five investigations and a patent that showed that external plant treatments with naked dsRNAs led to downregulation of plant endogenous genes, including silencing of the 3-phosphate synthase (*EPSPS*) gene in tobacco and amaranth leaves [28], *MYB1* gene in the orchid flower buds [29], *Mob1A*, *WRKY23*, and *Actin* genes in *Arabidopsis* and rice [30], two sugar transporter genes *STP1* and *STP2* in tomato seedlings [31], a downy mildew susceptibility gene *LBDIf7*, and a glutathione S-transferase *GST40* gene in grapevine [32,33]. According to the data, external plant dsRNA treatments led to the dsRNA uptake, reduced mRNA levels of the gene targets, and some phenotypic or biochemical changes.

We also found that there were quite limited studies where RNA-seq or other approaches would be applied to analyze the processing of exogenous dsRNAs applied to plant surfaces, while such studies are required to uncover the molecular mechanisms of exogenously induced RNAi. Several studies performed RNA-seq to detect virus-specific sRNAs occurring in plant tissues with and without dsRNA treatments [11,18] or to analyze the sRNA population in plant leaves treated with dsRNA targeting fungal genes [8,20,34]. Using RNA-seq, Uslu et al. [35] found that naked *GFP*-dsRNAs and *GFP*-hpRNAs did not induce *GFP* transgene silencing in *N. benthamiana* and were not processed into siRNAs. However, high-pressure spraying of *GFP*-encoding synthetic siRNAs was efficient in inducing *GFP* silencing and *GFP*-siRNA amplification through the process of transitivity in *N. benthamiana* according to RNA-seq analysis [12,17]. To the best of our knowledge, there were no studies analyzing dsRNA processing and post-treatment siRNA fraction after exogenously induced silencing of a plant endogenous gene. Such studies would allow the development of promising tools to manage plant gene expression for crop improvement and plant gene functional studies.

In this study, we aimed to analyze sRNAs occurring after plant treatments with dsRNAs targeting the gene of chalcone synthase (CHS), the key enzyme in the flavonoid/anthocyanin biosynthesis pathway, in Arabidopsis. Recently, we demonstrated that exogenous dsRNA and siRNA can efficiently downregulate mRNA levels of several genes involved in anthocyanin biosynthesis in *A. thaliana*, including the *AtCHS* gene, *AtMYBL2* gene encoding an R3-type single-MYB protein, and *AtANAC032* gene encoding a NAC-type transcription factor [12]. The plants treated with the *AtCHS*-specific dsRNAs exhibited lowered anthocyanin content and lighter leaf color in comparison with *A. thaliana* treated with water or *NPTII*-dsRNA, while the plants treated with dsRNAs targeting the *MYBL2* and *ANAC032* transcriptional repressors of anthocyanin biosynthesis accumulated higher levels of anthocyanins and showed darker leaf color [12]. In the present study, we showed that plant foliar treatments with both *AtCHS*- and *NPTII*-dsRNAs led to the emergence of a large number of *AtCHS*- and *NPTII*-specific sRNAs that have not been detected after control water treatment. The data revealed that the exogenous *AtCHS*-encoding dsRNA downregulated mRNA levels of the *AtCHS* gene and was processed into specific siRNAs, while *NPTII*-dsRNA did not induce *AtCHS* silencing and was presumably degraded. Results of the present study highlight a significant potential of exogenous dsRNAs as a promising strategy to induce RNAi-based downregulation of plant gene targets for plant management and gene functional studies.

## 2. Results

**Figure 1:**
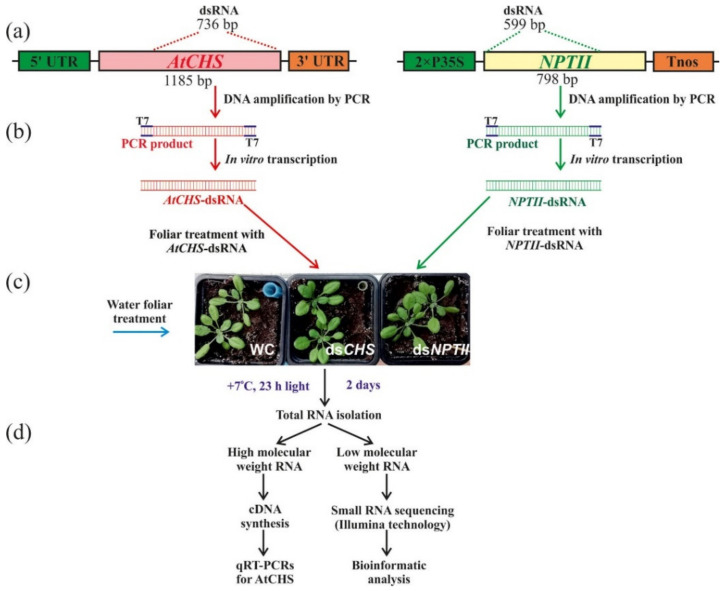
Schematic representation of the experiment conducted in the study to verify the effects of external dsRNA treatments in *Arabidopsis thaliana* on small RNAs profiles and *AtCHS* gene expression. (**a**) Representation of *AtCHS-* and *NPTII*-coding regions with positions of the *AtCHS*- and *NPTII*-specific dsRNA; (**b**) PCRs with the T7 promoter appended to both PCR primers for *AtCHS* and *NPTII* partial amplification, followed by in vitro dsRNA production; (**c**) plant foliar treatments with *AtCHS*- and *NPTII*-specific dsRNA; (**d**) low- and high-molecular-weight RNA isolation and processing. 2xP35S—the double 35S promoter of the cauliflower mosaic virus (CaMV); *AtCHS*—the chalcone synthase gene from *A. thaliana*; *NPTII*—the neomycin phosphotransferase II (*NPTII*) gene; Tnos—nopaline synthase terminator. T7–T7 promoter.

To analyze the effect of exogenous dsRNAs on the composition sRNA population, PCR and the in vitro transcription protocol were used to produce dsRNA of the *CHS* gene of *A. thaliana* and the bacterial *NPTII* gene. We synthesized the nonrelated *NPTII*-dsRNAs to verify whether any observed influence of exogenous dsRNAs on *AtCHS* mRNA levels and sRNA profiles were sequence specific. Large fragments of *AtCHS* cDNA and *NPTII* gene were amplified by PCR (Figure 1a). The obtained PCR products, containing T7 promoters at both ends, were used as templates for in vitro transcription (Figure 1b). For external application, the synthesized *AtCHS*- and *NPTII*-dsRNAs were diluted in water to a final concentration of 0.35 µg/µL. The *AtCHS*- and *NPTII*-dsRNAs (100 µL of each dsRNA per individual plant, i.e., 35 µg) or water (100 µL of sterile filtered water per individual plant) were applied on the leaf surface (Figure 1c) on both the adaxial and abaxial sides of four-week-old rosettes of *A. thaliana* by spreading with sterile individual soft brushes [12,36]. Two whole four-week-old rosettes of *A. thaliana* were treated per each type of exposure: water (rosettes WC-1a, WC-1b), *AtCHS*-dsRNA (rosettes ds*CHS*-3a, ds*CHS*-3b), and *NPTII*-dsRNA treatment (rosettes *NPTII*-4a, *NPTII*-4b) (Figure 1c). Then, we incubated the treated *A. thaliana* rosettes under anthocyanin-inducing conditions (+7 °C, and 23 h light) for two days, in order to induce *AtCHS* expression and anthocyanin biosynthesis, since under standard cultivation conditions, *AtCHS* mRNA and anthocyanin levels were low [12].

Importantly, we treated the four-week-old rosettes of *A. thaliana* at a late day time (21:00–21:30) under low soil moisture conditions, since appropriate plant age, late day time, and low soil moisture (at the time of dsRNA application) were important parameters for successful gene suppression in transgenic *A. thaliana* according to our analysis [12,36]. An analysis of the dsRNA concentration effect on transgene silencing in *A. thaliana* was performed previously [9], indicating that 35 µg of a transgene-encoding dsRNA resulted in the highest transgene silencing efficiency as compared to other dsRNA concentrations. Then, high-molecular-weight (HMW) and low-molecular-weight (LMW) RNA fractions were isolated two days after the dsRNA treatment to study whether exogenous application of the naked *AtCHS* and *NPTII*-dsRNAs on the foliar surface of wild-type *A. thaliana* could lead to any changes in the mRNA transcript levels of *AtCHS* gene and sRNAs in comparison with the control water treatment (Figure 1d). HMW RNA was used for cDNA synthesis and qRT-PCR analysis of the *AtCHS* gene expression, while LMW RNA was used for small RNA sequencing using Illumina technology.

**Figure 2:**
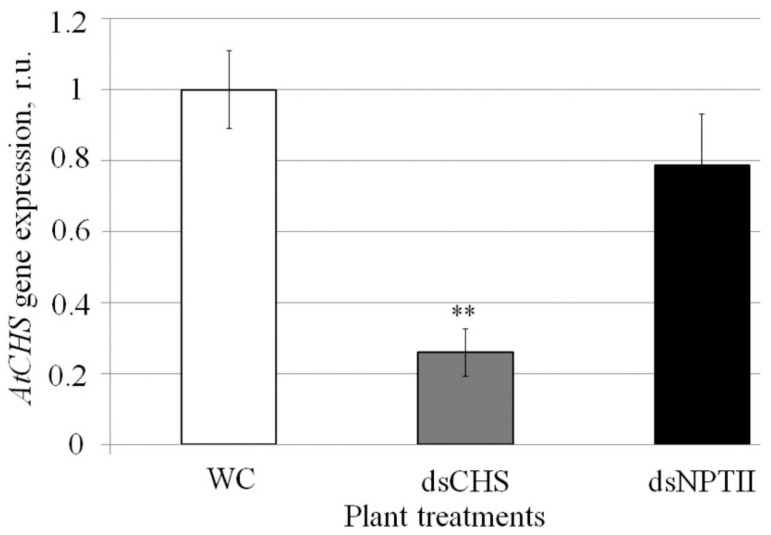
The effect of external *AtCHS*- and *NPTII*-encoding dsRNAs on *AtCHS* mRNA level in *Arabidopsis thaliana* analyzed by quantitative real-time PCR. WC—*A. thaliana* treated with sterile water; dsCHS—*A. thaliana* treated with *AtCHS*-dsRNA; dsNPTII—*A. thaliana* treated with *NPTII*-dsRNA; dpt—days post-treatment. *A. thaliana* plants were grown under anthocyanin-inducing (+7 °C, 23 h light) conditions for two days after treatment with sterile water or synthetic dsRNA. qRT-PCR data are presented as the mean ± SE. **—significantly different from WC at *p* ≤ 0.01 according to Student’s *t*-test.

First, we analyzed whether exogenous *AtCHS* and *NPTII*-dsRNAs affect mRNA levels of the *AtCHS* gene (Figure 2). Using qRT-PCR, we showed that the exogenous plant treatment with *AtCHS*-dsRNA significantly decreased *AtCHS* mRNA levels by four times, while the nonrelated *NPTII*-dsRNA did not significantly affect *AtCHS* expression (Figure 2).

**Figure 3:**
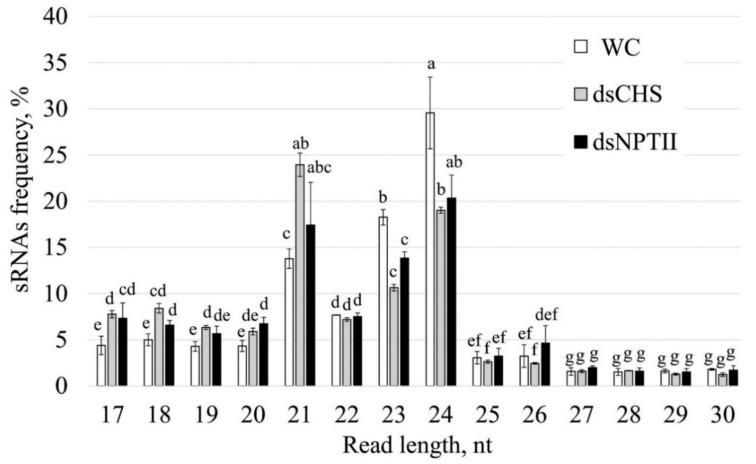
Length size distribution of total small RNAs in *Arabidopsis thaliana* after treatment with water or dsRNA. WC—*A. thaliana* plants treated with sterile water; dsCHS—*A. thaliana* treated with *AtCHS*-dsRNA; dsNPTII—*A. thaliana* treated with *NPTII*-dsRNA. The data are presented as the mean ± SE. Means followed by the same letter were not different using Student’s *t*-test. *p* < 0.05 was considered statistically significant.

Next, using the Illumina NovaSeq 6000 instrument, we obtained six RNA-seq libraries: two for water-treated plants, two for *AtCHS*-dsRNA-treated plants, and two for *NPTII*-dsRNA-treated plants (Table 1). In total, we obtained 526,174,120 reads, including 168,404,990 reads for the water-treated plants, 198,443,550 reads for *AtCHS*-dsRNA-treated plants, and 159,325,580 reads for *NPTII*-dsRNA-treated plants. After removing low-quality reads and unqualified reads (adapters, reads shorter than 17 and longer than 30 nucleotides, nongenomic reads (except for *NPTII*), ribosomal and chloroplast DNA sequences), 5.9–14.1 million clean reads were obtained for each sample (Table 1). Subsequently, the length of the sRNAs in each sample was determined. The distribution of the total sRNA population in length is shown in Figure 3. In all samples, 21-nt, 23-nt, and 24-nt sRNAs exhibited the highest abundance, while the frequency of other sRNA size classes was considerably lower. We also noticed that plant treatment with *AtCHS*-dsRNA resulted in a considerably increased level of 21-nt sRNAs, while the content of 23-nt and 24-nt sRNAs was in turn decreased in comparison to other treatments (Figure 3). After preprocessing, we obtained 56,944,787 reads, which were about 11% of the initial number. As a result, for further analysis, we used 25,247,201 reads for water treatment, 12,357,084 reads for ds*CHS* treatment, and 19,340,502 reads for ds*NPTII* treatment.

**Figure 4:**
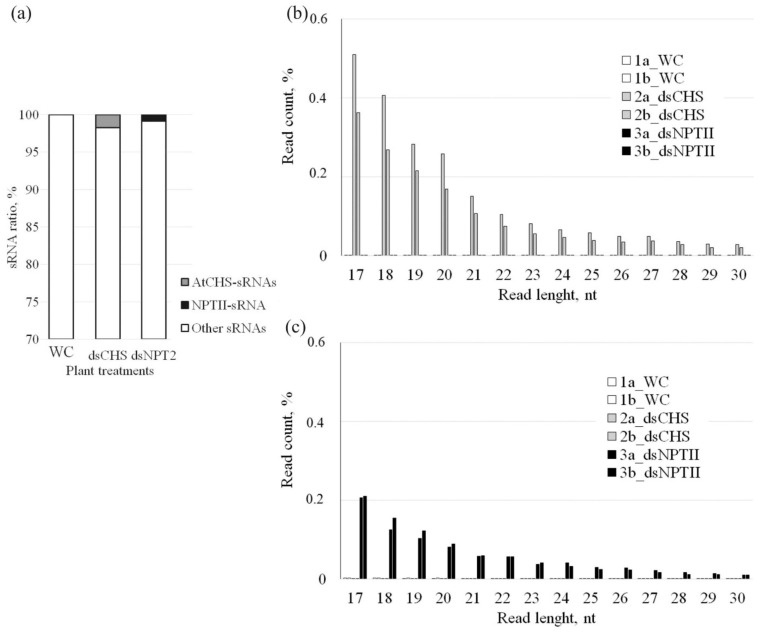
The effect of *AtCHS*-dsRNA and *NPTII*-dsRNA on the proportion and length size distribution of *AtCHS*-encoding 17–30-nt sRNAs among all detected small RNAs. (**a**) The proportion of *AtCHS*- and NPTII-encoding 17–30-nt sRNAs among all detected small RNAs. WC—*A. thaliana* plants treated with sterile water; dsCHS—*A. thaliana* treated with *AtCHS*-dsRNA; dsNPTII—*A. thaliana* treated with *NPTII*-dsRNA; (**b**) length size distribution of *AtCHS*-encoding 17–30-nt sRNAs; (**c**) length size distribution of *NPTII*-encoding 17–30-nt sRNAs. 1a_WC, 1b_WC—two *A. thaliana* plants treated with sterile water; 2a_dsCHS, 2b_dsCHS—two *A. thaliana* plants treated with *AtCHS*-dsRNA; 3a_dsNPTII, 3b_dsNPTII—two *A. thaliana* plants treated with *NPTII*-dsRNA.

Then, we analyzed the abundance and composition of 17–30-nt sRNAs aligned to the *AtCHS* and *NPTII* sequences (Figure 4). The analysis revealed that there were 1.75% of *AtCHS-* specific sRNAs and 0.85% of *NPTII*-specific sRNAs in *A. thaliana* treated with the corresponding dsRNAs among all obtained reads after preprocessing, while there were no such sequences in the water-treated control plants (Figure 4a). The data revealed that the length size distribution of the sRNAs mapping to *AtCHS* or *NPTII* (Figure 4) was different from the length size distribution of the total sRNA fraction (Figure 3). A large fraction of the *AtCHS-* or *NPTII-*specific sRNAs was presented by small-sized sRNAs ranging from 17 to 20 nt and reached more than half of all target-specific sRNAs (Figure 4). The data revealed that the shorter the length of both the *AtCHS-* or *NPTII-*specific sRNAs, the more read counts were detected, and the proportion of the *AtCHS-* or *NPTII-*specific sRNAs gradually decreased from the 17-nt sRNAs to 30-nt sRNAs.

**Figure 5:**
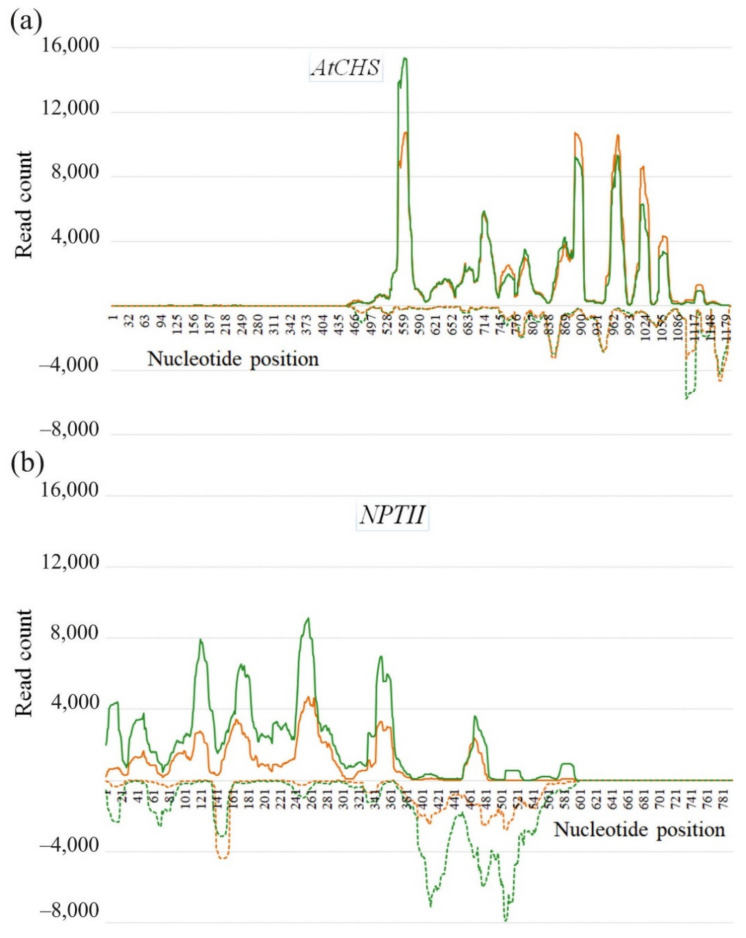
Strand-specific distribution of the 17–30-nt *AtCHS-* and *NPTII-*encoding sRNAs detected in *Arabidopsis thaliana* after dsRNA treatments along the *AtCHS* (**a**) and *NPTII* (**b**) gene coding regions. Read depth was counted by the number of reads on each position. Sense strand is plotted with a solid line above the x-axis, and antisense strand is plotted with a dotted line below the x-axis. Green and orange colors indicate two biological replicates.

We also analyzed the sRNA distribution patterns across both the *CHS* and *NPTII* genes (Figure 5a,b). The majority of sRNAs were not uniformly mapped along both the *CHS* and *NPTII* genes and covered the gene sequences unevenly with high frequencies of reads at particular positions, including 6–8 peak read count positions for the sense strand and 5–6 peak read count positions for the antisense strand. The *AtCHS* gene sense sequence was covered by 8 peaks of read counts (528–559 nt, 714–745 nt, 745–776 nt, 776–807 nt, 869–900 nt, 962–993 nt, 993–1024 nt, 1055–1086 nt), while the antisense sequence was covered by 6 peaks (776–807 nt, 838–869 nt, 931–962 nt, 1086–1117 nt, 1148–1179 nt) (Figure 5a). Similarly, the *NPTII* sense sequence was covered by 7 peaks (1–31 nt, 31–61 nt, 91–121 nt, 151–181 nt, 241–271 nt, 331–361 nt, 451–481 nt). In the mentioned intervals, the number of reads prevailed over read counts at other positions. For example, there are almost no sRNAs mapping to the sense *NPTII* strand at 391–451 nt position (Figure 5a). The antisense sequence of the *NPTII* gene was covered by a fewer number of peaks with 5 peaks of read counts (1–31 nt, 61–91 nt, 121–151 nt, 391–421 nt, 481–511 nt). Notably, the peak positions on the sense strand did not coincide with the peak positions on the antisense strand (Figure 5). Most reads mapped to the sense strands of both the *AtCHS* and *NPTII* genes. For the *AtCHS* gene, there were 1.4–1.5 times more sequences identical to the sense than for the antisense gene strand (Figure 5a). Similarly, for the *NPTII* gene, reads mapped to the sense strand 2.5–2.7 times higher than to the antisense *NPTII* strand (Figure 5b).

## 3. Discussion

The increasing human population and discussions about the safety of transgenic plants promote the development of new strategies to regulate plant properties without genomic manipulations. Plant foliar treatment with dsRNA has considerable potential as an innovative approach for gene regulation in plants and for plant pathogen control to ensure low-risk and environmentally friendly plant management. Recently, we demonstrated that foliar treatments of *A. thaliana* with gene-specific dsRNAs and siRNAs significantly reduced expression of the target genes, including *AtCHS* and two genes *AtMybL2* and *AtANAC032* encoding transcriptional repressors of anthocyanin biosynthesis [12]. In this study, before sRNA-seq, we analyzed *AtCHS* mRNA levels and confirmed that *AtCHS* mRNA levels sharply decreased after external plant treatments with the *AtCHS*-specific dsRNA, while *AtCHS* expression was not considerably affected after application of the nonspecific *NPTII*-dsRNA.

In the current literature, there is a lack of data on the plant uptake and processing of exogenously applied dsRNAs. Using confocal microscopy and some other approaches, several investigations have shown that exogenous dsRNA is taken up by the plant and is transferred via the plant vascular system [8,12,14,15,37]. According to the studies, these dsRNAs have been systemically translocated within the plant and were also detectable inside infecting fungal pathogens or insect pests [8,10]. However, the mechanism of uptake of exogenously applied dsRNAs and siRNAs still remains unclear. Song et al. [34] found that exogenous dsRNA was absorbed more efficiently via the wounded than the healthy surface of wheat coleoptiles. Based on their microscopic studies, the authors proposed that the used dsRNA entered the damaged cells of the wounded coleoptile surface and was transferred via the tracheary elements. Confocal microscopy revealed that fluorescently labeled dsRNAs applied onto the plant leaves can be detected in leaf veins, parenchyma cells, and companion cells, as well as in trichomes and stomata [8,12]. Therefore, there is a possibility of dsRNA stomatal uptake in plants. It is likely that externally applied synthetic dsRNAs and siRNAs could enter plant tissues and cells, exploiting the same natural mechanisms as extracellular nucleic acids originating from plant microbial pathogens, insects, or viruses. Pathogenesis-related extracellular RNA and DNA derived from plant microbial and virus pathogens are known to induce plant innate immunity and regulate self- and non-self-recognition in plants [38,39,40,41,42]. It is proposed that extracellular pathogen- and virus-related DNA and RNA molecules are perceived as genuine microbe- and pathogen-associated molecular patterns (MAMPs and PAMPs) in plants and act via pattern recognition receptors (PRRs) to induce pattern-triggered plant immune (PTI) signaling. However, there are again scarce data on the plant perception of pathogenesis-related DNA and RNA. To date, no specific receptors responsible for the recognition and uptake of extracellular DNA and RNA have been identified in plants. The findings by Niehl et al. [43] shed light on this issue, revealing that applied virus-related dsRNA induced PTI responses in Arabidopsis via a somatic embryogenesis receptor-like kinase 1 (SERK1), and not through antiviral DICER-like proteins (DCL). This study suggested that dsRNA-mediated PTI involved membrane-associated processes and operated independently of RNA silencing.

According to Uslu et al. [35], naked *GFP*-dsRNAs and *GFP*-hpRNAs did not induce *GFP* silencing in *N. benthamiana* and were not processed into siRNAs, indicating insufficient dsRNA uptake by plant cells. However, high-pressure spraying of *GFP*-encoding synthetic siRNAs was efficient in inducing *GFP* silencing in *N. benthamiana* [14,17]. Uslu et al. [17] performed RNA-seq to investigate the profile and amounts of sRNAs mapping to *GFP* after exogenous *N. benthamiana* treatments with three synthetic 22-nt siRNAs. According to the study, the 22-nt siRNA targeting the 3′ of the *GFP* transgene was less efficient in inducing silencing when compared with the siRNAs targeting the 5′ and middle region of the *GFP*. Application of the siRNAs induced *GFP* silencing and *GFP*-siRNA amplification through the process of transitivity. It has been shown that dsRNA treatments considerably lowered viral-derived sRNAs and improved plant virus resistance [11,18]. However, the profile of sRNAs derived from externally added virus-specific dsRNAs (not from infecting virus) still requires investigation. The sRNA-seq analysis of plant tissues treated with fungal-derived dsRNA revealed a passage of the dsRNA via the plant vascular system and processing into siRNAs by the fungal DICER-LIKE 1 after uptake by the pathogen [8,20].

Although a number of studies have demonstrated that exogenous gene-specific dsRNAs can induce downregulation of target plant endogenous genes [28,29,30,31,32,33], there are no studies (to the best of our knowledge) where RNA-seq would be applied to analyze post-application processing of exogenous dsRNAs targeting plant genes. In the present study, we firstly showed that foliar application of both *AtCHS*- and *NPTII*-dsRNAs led to the emergence of a large number of sRNAs mapping to the *AtCHS* and *NPTII*, which has not been detected after the control water treatments. The data indicate that exogenous *AtCHS*-dsRNAs are processed into siRNAs and eventually target the plant *AtCHS* gene leading to *AtCHS* downregulation, while *NPTII*-dsRNAs were presumably degraded. The prevailing frequencies of the 21-nt, 23-nt, and 24-nt sRNAs in the total sRNA fraction confirmed the previously obtained data of other scientists that the main part of the sRNA pool is represented by molecules ranging in size from 21 to 24 nt [4,5]. The length size distribution of the total sRNA pool after exogenous dsRNA application was also similar to the data by Power et al. [20], showing that 21-nt and 24-nt sRNAs prevailed. Notably, while there were three peaks in the sRNA amounts of 21-nt, 23-nt, and 24-nt sizes in the total sRNA fraction, we did not detect a considerable increase in the frequency of certain sizes of the *AtCHS*- and *NPTII* sRNAs. The length size distribution of the *AtCHS*- and *NPTII*-derived sRNAs was similar to that reported by Koch et al. [8] for *CYP3*-dsRNA-derived sRNAs in terms of the distribution of sRNA levels gradually decreasing from small-sized sRNAs to longer ones. This distribution of sRNA lengths suggested that the dsRNA-derived sRNAs may gradually degrade, leading to this gradually decreasing sRNA length. Thus, the exogenously applied dsRNAs might be processed according to different laws as compared with the naturally formed dsRNAs, which are processed by DICER to 21–24-nt siRNAs. For externally applied dsRNA, it is possible that a significant portion of the dsRNA is destroyed nonsystematically, because there is no specific sRNA size fraction, and the smallest sRNA size prevails. The present study revealed the *CHS*- and *NPTII*-specific sRNAs unevenly distributed across both the *CHS* and *NPTII* genes, with peaks of read counts at particular positions on both sense and antisense strands. Koch et al. [8] also detected uneven distribution of *CYP3*-dsRNA-derived sRNAs mapping to *CYP3*-dsRNA sequence. Further studies are needed to investigate whether this irregular sRNA mapping pattern is a result of rapid degradation of certain sRNAs or whether the read count peaks represent hotspots of secondary siRNAs generated by a transitivity-related process.

Taken together, the data show that exogenous dsRNAs are processed into siRNAs whose functional fraction induces RNAi and downregulates mRNA levels of the target gene. The results reported in this work highlight the potential of exogenous dsRNA application for silencing of specific plant genes and modulating desired plant traits.

## 4. Materials and Methods

### 4.1. Plant Material and Growth Conditions

The seeds of wild-type *A. thaliana* (cv. Columbia) were vapor-phase sterilized and plated on solid ½ Murashige and Skoog (MS) medium as described [16]. The plates were placed at 22 °C for 1 week in a growth chamber (Sanyo MLR-352, Panasonic, Osaka, Japan) at a light intensity of ~120 μmol m^−2^ s^−1^ over a 16 h daily light period. Then, 1-week-old seedlings of *A. thaliana* were planted in pots 7 cm × 7 cm containing 100 g of commercially available rich soil “Universal Soil” (Fasko, Moscow, Russia). The soil consisted of riding peat, lowland peat, sand, limestone (dolomite) flour, and complex mineral fertilizer with microelements. The content of nutrients available to plants (mg/kg) was not less than: nitrogen—350; phosphorous—400; potassium—500; pH—6–7. The soil was well irrigated by filtered water applied at the bottom of the pots. Then, the plants were grown in the chamber at 22 °C under plastic wrap for additional three weeks without additional irrigation. Plant dsRNA treatments were performed using four-week-old rosettes of *A. thaliana*. After the RNA treatments, the four-week-old plants were incubated for additional seven days under anthocyanin-inducing (+7 °C, 23 h daily light period) conditions in a growth chamber (KS-200, Smolenskoe SKTB SPU, Smolensk, Russia) without further irrigation to induce *AtCHS* expression and anthocyanin accumulation.

### 4.2. Isolation and Sequencing of AtCHS mRNA Transcripts

Full-length coding cDNA sequences of *AtCHS* (AT5G13930.1, 1188 bp) were amplified by RT-PCR using total RNA samples extracted from the adult leaves of *A. thaliana* as described [12]. The RT-PCR products were subcloned into pJET1.2/blunt and sequenced as described previously [9].

### 4.3. dsRNA Synthesis and Application

The *AtCHS*- and *NPTII*-dsRNAs were synthesized using the T7 RiboMAX™ Express RNAi System (Promega, Madison, WI, USA). For this purpose, a large cDNA fragment of *AtCHS* (736 bp out of 1188 bp) was amplified by PCR for in vitro transcription and dsRNA production. We amplified a large fragment of *NPTII* (GenBank AJ414108, 599 bp out of 798 bp) using pZP-RCS2-nptII plasmid [44]. The T7 promoter sequence was introduced into both the 5′ and 3′ ends of the amplified *AtCHS* or *NPTII* in a single PCR for each gene using primers listed in Table S1 (Figure 1). The PCRs were performed in the Bis-M1105 Thermal Cycler programmed according to T7 RiboMAX™ Express RNAi System instructions. PCR was carried out in a final volume of 30 µL, containing 1X Taq reaction buffer with 3 mM MgCl_2_, 0.5 µL plasmid DNA (50 ng), 200 µM dNTPs, 0.2 µM of each primer, and 2.0 units of Taq DNA polymerase (Evrogen, Moscow, Russia). After an initial denaturation at 95 °C for 5 min, the first 5 cycles were performed as follows: 95 °C for 10 s, 63 °C (*AtCHS*) or 65 °C (*NPTII*) for 10 s, 72 °C for 38 s (*AtCHS*) or 38 s (*NPTII*), followed by 40 cycles each of 95 °C for 10 s and 72 °C for 48 s (*AtCHS*) or 48 s (*NPTII*). After a final extension at 72 °C for 5 min, PCR fragments were loaded on 1% agarose gel and purified by the Cleanup Standard kit (Evrogen). Then, the obtained PCR products were used as templates for in vitro transcription and dsRNA synthesis following the manufacturer’s protocol. The resultant dsRNAs were analyzed by gel electrophoresis and spectrophotometry to estimate dsRNA purity, integrity, and amount.

The *AtCHS*- and *NPTII*-dsRNAs were applied to the foliar surface of four-week-old rosettes of wild-type A. thaliana by spreading with individual soft brushes (natural pony hair) sterilized by autoclaving (Figure 1) as described [12,36]. For each dsRNA treatment, 35 µg of the dsRNA were diluted in 100 µL of nuclease-free water and applied to all leaves of one rosette on both the adaxial and abaxial sides. Two rosettes were treated in total per each type of condition (35 µg of dsRNA per each rosette). The water and dsRNA treatments were performed at a late day time (21:00–21:30) under low soil moisture conditions using four-week-old rosettes of *A. thaliana*, since the appropriate late day time, low soil moisture, and plant age at the time of dsRNA application were important parameters for successful gene suppression in *A. thaliana* according to our recent analysis [12,36]. Soil water content before dsRNA treatment was 50–60%.

### 4.4. RNA Isolation

For RNA isolations, two whole four-week-old rosettes of *A. thaliana* were collected two days after treatments per each type of treatments: water treatment (rosettes WC-1a, WC-1b), *AtCHS*-dsRNA treatment (rosettes ds*CHS*-3a, ds*CHS*-3b), and *NPTII*-dsRNA treatment (rosettes *NPTII*-4a, *NPTII*-4b). High-molecular-weight (HMW) and low-molecular-weight (LMW) RNA fractions were isolated using a modified cetyltrimethylammonium bromide (CTAB)–lithium chloride (LiCl)-based protocol [45]. First, we began isolation of total RNA using the CTAB-polyvinylpyrrolidone K30 (PVP) extraction buffer prepared as described [45]. Tissue samples of *A. thaliana* (a whole 4-week-old rosette of 70–80 mg) were homogenized in the extraction buffer and incubated for 5 min at 65 °C in a water bath. After the above incubation, 700 µL of chloroform was added. The samples were then centrifuged at full speed (13,200 rpm, +4 °C) for 15 min (5415R, Eppendorf, Germany). Approximately, 1 mL of the water phase was mixed with 250 µL of 10 M LiCl (Panreac, Spain) and incubated at +4 °C overnight. After the above incubation, the samples were centrifuged at full speed (13,200 rpm, +4 °C) for 15 min.

To separate HMW RNAs, the pellets were air-dried and dissolved in 100 µL of sterile distilled water for the following precipitation with a 2.5 volume of precooled absolute ethanol at −20 °C overnight. Then, the HMW RNA samples were centrifuged at full speed (13,200 rpm, +4 °C) for 15 min. The air-dried pellets were resuspended in an appropriate volume of sterile distilled water and used for complementary DNA (cDNA) preparation.

After LiCl precipitation, 400 µL of the resulting upper aqueous phase was transferred into another clean 1.5 mL collection tube to separate LMW RNAs. The LMW RNAs were precipitated with 1/10 volume of 3 M sodium acetate (pH 5.2) and 2.5 volume of precooled absolute ethanol at −20 °C overnight.

### 4.5. Reverse Transcription and qRT-PCRs

cDNAs were synthesized using 1.5 µg of total RNA as described [46]. The 1 µL samples of reverse transcription products were then amplified by PCR and verified in the absence of DNA contamination using primers for the *AtCHS* gene (AT5G13930.1) listed in Table S1. The qRT-PCRs were performed with SYBR Green I Real-Time PCR dye and a real-time PCR kit (Evrogen, Moscow, Russia) as described [47] using two internal controls (GAPDH and UBQ) selected in previous studies as relevant reference genes for qRT-PCRs on Arabidopsis [48]. Amplification was carried out in a DTprime real-time thermocycler (DNA Technology, Moscow, Russia). PCR conditions consisted of 2 min at 95 °C, followed by 50 cycles of 10 s at 95 °C and 25 s at 62 °C. The expression was calculated by the 2^−ΔΔCT^ method [49]. The data were processed with the RealTime_PCR v.7.3 (DNA Technology, Russia). All gene identification numbers and used primers are listed in Table S1.

### 4.6. Small RNA Sequencing

The ethanol-precipitated LMW RNA fractions were sent for high-throughput sequencing using Illumina technology to Evrogen (Moscow, Russia). Incoming sample quality control was performed by horizontal electrophoresis in agarose gel. RNA was prepared (100 ng per biological replicate) for sequencing using the Qiagen Small RNA Sample Prep kit (Qiagen, Dusseldorf, Germany). The quality of the obtained libraries was checked using Fragment Analyzer (Agilent, CA, USA). Quantitative analysis was performed by qPCR. After quality control and DNA quantity estimation, the library pool was sequenced on an Illumina NovaSeq 6000 instrument (1 × 100 bp single-read sequencing). When sequencing cDNA libraries, the loading concentration was 300 pM, and the volume was 27 µL in accordance with the standard Illumina recommendations. The FASTQ files were obtained using bcl2fastq Conversion Software v2.20 (Illumina, San Diego, CA, USA). The recording format of the quality data string is Phred 33. As a result, 526,174,120 reads were obtained. General information about the read numbers is presented in Table 1. sRNA library sequences were deposited to the National Center for Biotechnology Information (NCBI) under Accession Number PRJNA827691 and in the database of the Laboratory of Biotechnology, Federal Scientific Center of the East Asia Terrestrial Biodiversity, Far Eastern Branch of the Russian Academy of Sciences, Russia (https://biosoil.ru/downloads/biotech/RNAseq/Arabidopsis/2021-03-20012551-data1(Our-RNAseq-1(2))/) (accessed on 8 May 2022).

### 4.7. Bioinformatic Analysis

Adaptor sequences (5′AACTGTAGGCACCATCAAT, 5′AGATCGGAAGAGCACACGT) were trimmed, and low-quality reads and reads shorter than 17 and longer than 30 nucleotides were removed from obtained high-throughput sequencing data with the BBDuk program [50]. Using the Bowtie program (0 mismatch available) [51], reads aligned to ribosomal and chloroplast DNA sequences and reads unaligned to the *A. thaliana* TAIR10 genome [52] were excluded from analysis (except for the *NPTII* gene). Then, using the same program, we analyzed the sRNAs aligned to *AtCHS* and *NPTII* sequences. Small RNA coverage data on *AtCHS* and *NPTII* gene sequences were obtained using BEDTools software [53]. The following data processing and line charts/bar graphs were plotted using Microsoft Excel based on only 17 to 30 nt long reads.

### 4.8. Statistical Analysis

The data are presented as mean ± standard error (SE) and were tested by paired Student’s *t*-test. The *p* < 0.05 level was selected as the point of minimal statistical significance in all analyses. Two whole four-week-old rosettes of *A. thaliana* were collected two days after treatments per each type of treatment (two biological replicates per each type of analysis).

## Figures and Tables

**Table 1:** Samples analyzed by sRNA-seq and read numbers obtained after high-throughput sequencing (Illumina NovaSeq 6000 instrument). WC—*A. thaliana* treated with sterile water; dsCHS—*A. thaliana* treated with *AtCHS*-dsRNAs; dsNPTII—*A. thaliana* treated with *NPTII*-dsRNA.

Plant Number	Treatment	Read Count	Reads after Preprocessing	FASTQ File Name
1a	WC	95,916,080	14,097,422 (14.7%)	1_CTATAC_L002_R1_001.fastq.gz
1b	WC	72,488,910	11,149,779 (15.4%)	4_TAATCG_L002_R1_001.fastq.gz
2a	dsCHS	96,022,164	5,250,614 (5.5%)	2_CTCAGA_L002_R1_001.fastq.gz
2b	dsCHS	102,421,386	7,106,470 (6.9%)	5_TACAGC_L002_R1_001.fastq.gz
3a	dsNPTII	49,357,278	5,928,333 (12%)	3_GACGAC_L002_R1_001.fastq.gz
3b	dsNPTII	109,968,302	13,412,169 (12.2%)	6_TATAAT_L002_R1_001.fastq.gz

## Data Availability

sRNA library sequences were deposited to the National Center for Biotechnology Information (NCBI) under Accession Number PRJNA827691 and in the database of the Laboratory of Biotechnology, Federal Scientific Center of the East Asia Terrestrial Biodiversity, Far Eastern Branch of the Russian Academy of Sciences, Russia (https://biosoil.ru/downloads/biotech/RNAseq/Arabidopsis/2021-03-20012551-data1(Our-RNAseq-1(2))/) (accessed on 8 May 2022).

## Supplementary Materials

The following supporting information can be downloaded at: www.mdpi.com/article/10.3390/ijms23105325/s1.
