# Supplementary material for: Exogenous dsRNA Induces RNA Interference of a Chalcone Synthase Gene in Arabidopsis thaliana
Source: Int J Mol Sci. 2022 May 10;23(10):5325. doi: 10.3390/ijms23105325 (PMC9142100; doi:10.3390/ijms23105325)
Supplement: Supplementary file 1 [file ijms-23-05325-s001.zip › ijms-1716319-supplementary.pdf]

Supplementary Table S1 Primers used in RT-PCR and qRT-PCRs.

Gene name (ID number)	Primer name	Primers, 5′-3′
Specific primers for dsRNA design, 5′-3′		
AtCHS (AT5G13930.1)	AtCHS-RNAs2<br>AtCHS- RNA-a2	5'TAATACGACTCACTATAGGGAGAGCTTCTTGGTCTCC GTCCTTCC<br>5'TAATACGACTCACTATAGGGAGATTAGAGAGGAACG CTGTGCAAG
NPTII (AJ414108,AY818 371)	npt-T71-s<br>npt-T72-a	5′TAATACGACTCACTATAGGGAGAATGTGGATTGAAC AAGATGGATTG<br>5′TAATACGACTCACTATAGGGAGATCCACCATGATATT CGGCAAGCAG
Primers for cDNA check-up on DNA contamination, 5′-3′		
AtGAPDH (GenBank NM_111283)	AtGapdh-s<br>AtGapdh-a	5′CTG GAA TGT CTT TCC GTG TC<br>5′ATT CGT TGT CGT ACC ATG AC
Primers for PCR and real-time PCR, 5′-3′		
AtCHS (AT5G13930.1)	AtCHS-Nach-S<br>AtCHS-realA	5′ATGGTGATGGCTGGTGCTTCTT<br>5′- CACATGGTTCTCAGGGTTAGC
NPTII (GenBank AJ414108)	nptII-realS<br>nptII-realA	5′TTGCTGAAGAGCTTGGCGGCGAAT<br>5′TCAGAAGAACTCGTCAAGAAGG
AtGAPDH (GenBank NM_111283)	AtGapdh-real-s<br>AtGapdh-real-a	5′TTG GTG ACA ACA GGT CAA GCA<br>5′AAA CTT GTC GCT CAA TGC AAT
AtUBQ (GenBank NM_001084884)	AtUBQ-realS<br>AtUBQ-realA	5′GGCCTTGTATAATCCCTGATGAATAAG<br>5′AAAGAGATAACAGGAACGGAAACATAGT
